# Dr. Mossman, on Fever

**Published:** 1801-02

**Authors:** G. Mossman

**Affiliations:** Bradford


### *To the Editors of the Medical and Phyſical Journal.*

GENTLEMEN,

BY enquiry and diſcovery, the condition of man is improved; and by the continued application of talent and induſtry to the art of healing, it is to be expected that much will be atchieved. The old fabrics of phyſic will undergo a cloſe examination; the rubbiſh will be removed, and the valuable materials only will be ſuffered to remain: the myſterious pe-

[illegible]dantry of the antient ſchools, and the blind devotion to antient ſyſtems, will be doomed to contempt; and medical opinions will command veneration, not becauſe of their antiquity, but becauſe they are proved to be true. The profeſſors of medicine are now acquiring additional knowledge of the mechaniſm of the human ſyſtem; and by the aid of obſervation and experiment, they are purſuing inquiries which will lead, perhaps, more directly to the cure of diſeaſe. The true end of ſcience is the production of new powers, and the means of applying them to uſeful purpoſes; and the unfolding of powers, which will infallibly leſſen the accumulated maſs of human evil, is an object of high importance. The votaries of medicine then, ſhould never be idle; for induſtry or accident may eventually teach them to ſubdue maladies, which now elude every exertion of their art. There was a period when *ague* and *ſyphylis* were incurable: the antidote to both is now well known. It is certainly compatible with probability, and I believe it to be true, that Nature holds a remedy for every evil by which we are aſſailed; and, perhaps, the time is not far diſtant, when there will be a diſcovery of powers, which ſhall very generally obviate the fatality of *fever* and *pulmonary conſumption.* In the primary ſtages of *phthiſis*, the *digitalis* certainly approximates to a ſpecific; and, perhaps, when properly adminiſtered, or when aided by proper auxillaries, it is poſſible that the plant may operate as a curative agent, even in the advanced ſtages of that diſeaſe. *For fever there is no remedy.* In this iſland, at leaſt, there never has been any power employed, which could, with any certainty, arreſt the progreſs of *fever* for a ſingle day. If this obſervation be conſiſtent with univerſal experience, let it apologize for the freedom of the ſubſequent diſcuſſion: If the progreſs of intellect be daily productive of beneficial inventions, ſurely, a ſcience which embraces ſo large a portion of the fate of man, cannot be ſtationary. If the combined ravages of *fever* and *pulmonary conſumption* are deſtructive of more than half of thoſe who periſh by diſeaſe, it is very certainly an object of much moment to obviate their fatal tendency; and the accompliſhment of this end, ought alone to be the limit of inquiry. The extended uſe of that invaluable plant, which is found to govern the heart, and to exhibit powers ſingularly curative in certain ſtates of *pulmonary conſumption*, is a recent diſcovery; and is it impoſſible that there alſo exiſts a power completely ſubverſive of the phænomena of fever? This is an inquiry of extreme importance, and nothing can be loſt by purſuing it. It is known that the preſence of *fever* is conſtituted or indicated by an increaſed action of the ſanguiferous ſyſtem; but it is not known what is the the remote and exciting cause of this morbid action; nor is it known why it should be so permanent. It is, I believe, consistent with the knowledge of every physician, that brisk *evacuations* recurred to on the first accession of *fever*, will sometimes be subversive of the *febrile action*; but I am persuaded it is also true, when this period is gone by, that the practitioner cannot maintain any other character than that of an observer. Although there are various doctrines of the nature and cure of *fever*, yet, respecting its leading features, there is a very general coincidence of opinion; and the more obvious phænomena of *fever* have been so strikingly uniform, as to produce descriptions very exactly similar. I have already observed, that the operation of brisk *evacuants* has been sometimes found to be subversive of *febrile action*; but if those powers be ineffectually employed, the morbid catenation will continue for an unlimited period, in defiance of every effort of medical interference. During the continuance of *fever*, the physician may, in the character of an observer, direct what he conceives to be a salutary regimen; he may also, perhaps, be able to promote some of the natural discharges, or to correct them, if they be morbidly profuse; but this, I apprehend, is the extent of his power. For an indefinite number of days or weeks, a chain of morbid action will continue to be exhibited, till a *crisis* or *solution* of the *fever* is effected, by means of which we know nothing. This mode of the termination of *fever*, however, is by no means a constant appearance; for it very frequently happens, that the morbid action gradually rises to a certain degree, without any striking exacerbation of symptoms, and then begins to decline imperceptibly, till it entirely disappear. If those phænomena continue to be exhibited, unconnected with, and independent of, medical exertion, it is surely incorrect to speak of the *cure of fever*.

About twelve years ago I published Observations upon an Epidemic Fever, in which I employed, what may be denominated, the *Brunonian instruments*; and I was much gratified, by having the recovery of cases, apparently desperate, ascribed to my professional skill. Youth and inexperience persuaded me, that I was in possession of artillery very certainly destructive of *fever*. A few failures, however, soon afterwards occurred to confirm my unacquaintance with the nature of *fever*, and the inefficacy of my curative agents. I turned over the pages of authors, but they did not afford me satisfactory information on the subject: I again had recourse to my own observations; and by comparing what I read with what I saw, I became well persuaded, that *febrile action* was a phænomenon, neither understood, nor curable by the means then employed. I am here to be underſtood as ſpeaking of *idiopathic fever*, or *fever ariſing from contagion.* In conſulting the various diſſertations on *fever*, I found one author extolling one remedy, and another man giving praiſe to another; I employed all thoſe boaſted remedies ſingly; I varied them, and I combined them; *evacuants*, *refrigerants*, *diaphoretics*, *tonics*, *antiſceptics*, *ſtimulants*, and *anodynes*, had all an impartial trial; and under every mode of treatment, ſome of my patients periſhed, and ſome recovered; nor did the iſſue appear to be effected by any exertion of mine. The following general concluſions, however, then forced themſelves upon my notice; 1ſt. that the application of powers repreſſive of arterial action, was manifeſtly beneficial; and, 2dly. that the application of powers of a contrary tendency, was as manifeſtly injurious. I do admit, that ſome of the moſt diſtreſſing ſymptoms of *fever* may be alleviated, and ſometimes completely obviated by the inſtruments of medicine; yet, I very ſincerely believe, that could there be a juſt eſtimate of the good and the ill occaſioned by medical interference in *fevers*, I have no heſitation in aſſerting, that the balance will be found to be againſt our art. My experience has been too ample for me to miſtake; and I am well perſuaded, that a greater number of patients labouring under *continued fever*, will recover by being treated with *toaſt and water*, than by an exhibition of the remedies now in uſe.

We are not yet ſufficiently acquainted with the laws which direct the ſyſtem in a ſtate of health, and we know ſtill leſs of the circumſtances which influence it in a ſtate of diſeaſe; but I am convinced, that if the cure of *fever* be practicable, it is to be effected either *by exciting an action ſubverſive of the one preſent*, *or by directly arreſting the catenation of diſeaſe.* I believe it is true, that there are degrees of morbid action, and that a greater action is ſubverſive of an action leſs powerful. This doctrine is ſupported by authorities the moſt reſpectable: It has been obſerved, that one morbid action only can operate in the ſyſtem at the ſame time; and if two contagions be received, that the leſſer remains in a latent ſtate, till the greater either ceaſes to act, or is nearly exhauſted. It has further been obſerved, that the morbid action of the *plague* baniſhes from the city or place where it prevails, all inferior epidemic action. It is alſo true, that at the approach of the *yellow fever* in extreme malignity, all other epidemics inſtantly diſappear: it is conſiſtent too with the obſervations of every one, that the phænomena of *mania* and *pregnancy* will very conſtantly impede the progreſs of *pulmonary conſumption.* An additional ſpecification is unneceſſary; for, if what I have ſtated be true, it it is impoffible not to paufe! Thefe facts may afford ufeful information. The evidence refpecting the curative operation of *calomel* in the *yellow fever*, which prevailed in *Philadelphia*, is too ample to admit of doubt; and I am ftrongly inclined to believe, that the circumftance of its exciting a new action, will account for its beneficial effects. It is well known, that *mercury* is deftructive of the *fyphilitic virus*, and that as foon as the action of the former is apparent, the fymptoms of the latter begin to difappear. I know too, that by means of *mercury*, I can hold in check the action of the *variolous contagion*, and by the fame power render the difeafe, even in its worft form, very certainly mild: it is impoffible to know thofe facts, and not to draw inferences. I make little account of the fuppofed action of *calomel*, as an *evacuant*; for it is certainly true, that medicines poffeffing powers very ftrongly purgative, have been fruitlefly employed in checking the progrefs of *fever*; nor do I fubfcribe to any fuppofed chemical action of *mercury* on the fyftem of fluids; for, if that fuppofition be juft, there are means infinitely more eligible for the accomplifhment of the end propofed. There are, perhaps, other powers which may be fuccefsfully employed to fulfil my intention of the cure of *fever*; but I think it probable, that *mercury* will continue to hold the firft place. For the purpofe of directly arrefting the catenation of difeafe, I recommend the *digitalis*: the power of this plant over the action of the heart, is too well known to need any comment. The *fymptomatic fever* accompanying *pulmonary confumption*, is fo perfectly under the management of the *fox-glove*, that the phyfician may keep the patient's pulfe at any given number, and thereby be enabled to adminifter tonic or other remedies radically curative of the complaint.

I am now employed in making obfervations upon the fubject of curing *fever* by the means I have recommended, and I hope foon to be enabled to lay before you the refult of my experience. I have been too often difappointed to feel extremely fanguine; but if my future trials correfpond with the advantages which I think I have already gained, I am perfuaded that the practice will add fomething valuable to the treafury of medicine. I am,

GENTLEMEN,

Your's, moft refpectfully,

G. MOSSMAN.

*Bradford, Nov.* 24, 1800.

